# Somatostatin neuron contributions to cortical slow wave dysfunction in adult mice exposed to developmental ethanol

**DOI:** 10.3389/fnins.2023.1127711

**Published:** 2023-03-20

**Authors:** Donald A. Wilson, G. Fleming, C. R. O. Williams, C. M. Teixeira, J. F. Smiley, Mariko Saito

**Affiliations:** ^1^Nathan S. Kline Institute for Psychiatric Research, Orangeburg, NY, United States; ^2^Department of Child and Adolescent Psychiatry, New York University School of Medicine, New York, NY, United States; ^3^Department of Neuroscience and Physiology, New York University School of Medicine, New York, NY, United States; ^4^Department of Psychiatry, New York University School of Medicine, New York, NY, United States

**Keywords:** fetal alcohol spectrum disorder (FASD), somatostatin, slow wave sleep (SWS), prefrontal cortex, GABA, cortical interneurons, closed loop optogenetics

## Abstract

**Introduction:**

Transitions between sleep and waking and sleep-dependent cortical oscillations are heavily dependent on GABAergic neurons. Importantly, GABAergic neurons are especially sensitive to developmental ethanol exposure, suggesting a potential unique vulnerability of sleep circuits to early ethanol. In fact, developmental ethanol exposure can produce long-lasting impairments in sleep, including increased sleep fragmentation and decreased delta wave amplitude. Here, we assessed the efficacy of optogenetic manipulations of somatostatin (SST) GABAergic neurons in the neocortex of adult mice exposed to saline or ethanol on P7, to modulate cortical slow-wave physiology.

**Methods:**

SST-cre × Ai32 mice, which selectively express channel rhodopsin in SST neurons, were exposed to ethanol or saline on P7. This line expressed similar developmental ethanol induced loss of SST cortical neurons and sleep impairments as C57BL/6By mice. As adults, optical fibers were implanted targeting the prefrontal cortex (PFC) and telemetry electrodes were implanted in the neocortex to monitor slow-wave activity and sleep-wake states.

**Results:**

Optical stimulation of PFC SST neurons evoked slow-wave potentials and long-latency single-unit excitation in saline treated mice but not in ethanol mice. Closed-loop optogenetic stimulation of PFC SST neuron activation on spontaneous slow-waves enhanced cortical delta oscillations, and this manipulation was more effective in saline mice than P7 ethanol mice.

**Discussion:**

Together, these results suggest that SST cortical neurons may contribute to slow-wave impairment after developmental ethanol.

## 1. Introduction

Developmental ethanol exposure disrupts subsequent normal sleep structure in animal models (Stone et al., 1996; Criado et al., 2008; Volgin and Kubin, 2012; Wilson et al., 2016; Ipsiroglu et al., 2019) and humans (Pesonen et al., 2009; Jan et al., 2010; Wengel et al., 2011; Chen et al., 2012; Inkelis and Thomas, 2018). Given the critical role of sleep for synaptic homeostasis (Tononi and Cirelli, 2014), emotional regulation (Goldstein and Walker, 2014; Ben Simon et al., 2020), memory consolidation (Born et al., 2006; Stickgold and Walker, 2007; Diekelmann and Born, 2010), and many other basic neurocognitive functions, this developmental ethanol-induced sleep impairment could contribute to a variety of behavioral outcomes associated with fetal alcohol spectrum disorder (FASD). The sleep impairment can include reduced time in slow wave sleep, increased sleep fragmentation, reduced cortical slow wave (delta) amplitude, reduced sleep spindle density, and impaired sleep homeostasis following sleep deprivation (Stone et al., 1996; Criado et al., 2008; Troese et al., 2008; Wengel et al., 2011; Chen et al., 2012; Volgin and Kubin, 2012; Wilson et al., 2016; Inkelis and Thomas, 2018; Lewin et al., 2018; Ipsiroglu et al., 2019). These diverse aspects of sleep/wake physiology are under the control of diverse brain regions and diverse neuromodulatory systems. However, GABAergic inhibitory interneurons, across multiple brain regions also play an important role in many aspects of sleep physiology (Pace-Schott and Hobson, 2002; Saper et al., 2010; Adamantidis et al., 2019). For example, sleep-wake state changes and state maintenance are stabilized by hypothalamic and basal forebrain circuits wherein inhibitory interneurons suppress activity of the ascending reticular activating system while the subject is asleep and a different population of GABAergic neurons suppress sleep circuits while the subject is awake (Saper et al., 2010; Xu et al., 2015). In addition, inhibitory interneurons in the neocortex are involved in entraining delta band slow-wave oscillations that occur during non-REM, slow-wave sleep (Steriade et al., 1993b; Pace-Schott and Hobson, 2002; Funk et al., 2017). Large amplitude slow-wave oscillations are critical for memory consolidation, synaptic homeostasis, and metabolic waste clearance that occur during sleep (Knyazev, 2012; Harmony, 2013; Xie et al., 2013).

During slow wave sleep, slow (<1 Hz) cortical oscillations (Steriade et al., 1993b) are spontaneously initiated primarily in prefrontal cortex and travel as an anterior-posterior wave across the cortex (Massimini et al., 2004; Niethard et al., 2018; Timofeev et al., 2020). These slow waves help organize and synchronize other slow wave sleep events such as the large amplitude delta frequency band (1–5 Hz) oscillations characteristic of slow wave sleep and sleep spindles (Molle and Born, 2011; Staresina et al., 2015). Slow wave oscillation amplitude during sleep can be enhanced by applying reinforcing stimulation (Bellesi et al., 2014). For example, in humans, transcranial stimulation oscillating at <1 Hz using a variety of technical approaches can enhance slow wave and delta band power as well as the time spent in slow wave sleep (Marshall et al., 2006; Massimini et al., 2007; Ketz et al., 2018). Auditory stimulation applied in closed loop feedback with spontaneous slow waves (<1 Hz) can similarly enhance slow wave amplitude as well as sleep spindle power (Ngo et al., 2013). Importantly enhancing slow wave sleep with either technique enhances hippocampal-dependent memory (Marshall et al., 2006; Ngo et al., 2013; Henin et al., 2019). Similar stimulation-induced slow waves (Vyazovskiy et al., 2009) and closed loop modulation of slow wave oscillations and memory have been observed in rodents (Moreira et al., 2021).

One class of GABAergic cells involved in cortical sleep-related oscillations is somatostatin expressing (SST) neurons (Kuki et al., 2015; Xu et al., 2015; Yavorska and Wehr, 2016; Funk et al., 2017; Espinosa et al., 2019; Bugnon et al., 2022). In rodents, SST neurons in the neocortex help promote slow wave neural oscillations by inducing 100–200 ms of inhibition (i.e., a cortical down-state) following the excitation induced by thalamic and cortical excitation of pyramidal cells as shown both *in vivo* (Funk et al., 2017) and in computational modeling (Bugnon et al., 2022). Cortical SST neuron activity is phase locked to cortical slow wave oscillations (Funk et al., 2017). This cycling between excitation and inhibition is observed as slow-wave oscillations in slow-wave sleep EEG. Chemogenetic or optogenetic activation of neocortical SST neurons enhance cortical slow wave amplitude and slow wave sleep duration and the extensive anterior-posterior extent of cortical SST+ axons could promote slow-wave propagation across the cortex (Funk et al., 2017). Overall SST activity is reduced during slow-wave sleep, which has been suggested to result in disinhibition of parvalbumin-expressing interneurons to promote cortical sleep spindles (Niethard et al., 2018).

Developmental ethanol exposure has profound effects on GABAergic neurons in several brain regions (Skorput et al., 2015; Smiley et al., 2015), and we have previously reported that postnatal day 7 (P7) ethanol exposure significantly reduces SST neuron number in neocortex through adulthood (Smiley et al., 2019). Furthermore, P7 ethanol can reduce time spent in slow-wave sleep (Wilson et al., 2016; Lewin et al., 2018), enhances sleep fragmentation (increased state transitions) (Wilson et al., 2016; Lewin et al., 2018), and reduces delta band oscillation amplitude in neocortex (Apuzzo et al., 2020). Here we began to explore whether the loss of SST GABAergic neurons in the neocortex could contribute to the changes in adult mice slow-wave activity known to be induced by developmental ethanol exposure. As a starting point, we focused on a single important variable out of the myriad factors involved in sleep physiology and sleep function. We used optogenetic activation of spared SST neurons in ethanol exposed and saline control mice to manipulate cortical slow-wave activity (Funk et al., 2017; Bugnon et al., 2022). Identifying mechanisms of developmental ethanol-induced sleep impairments is a first step toward cell-targeted treatment.

## 2. Materials and methods

### 2.1. Subjects

Male and female SST-Cre (Jackson stock 013044) × Ai32 (Jackson stock 024109) mice were used. These mice express channelrhodopsin-2/EYFP in all SST neurons (Madisen et al., 2012). At postnatal-day seven (P7) mice were injected subcutaneously in the back with ethanol (2.5 g/kg), while littermate controls were injected with the same volume of saline solution as previously described (Olney et al., 2002; Saito et al., 2007). All mice in a litter were randomly assigned to either ethanol or saline treatment. Pups were injected twice, 2 h apart and immediately returned to the home cage after each injection. This P7 ethanol treatment protocol induces a peak truncal blood alcohol level of 0.5 g/dL (i.e., highest level observed within 6 h of the second injection) as analyzed with an Alcohol Reagent Set (Pointe Scientific, Canton, MI, USA) (Saito et al., 2007). This is a commonly used model of late term binge ethanol exposure that allows ethanol exposure at a precise developmental point, allows littermate controls, and does not impact the maternal physiology (Olney et al., 2002; Saito et al., 2007; Wilson et al., 2016). Pups were weaned at P28 and then housed (cage width × length × height:18 × 28 × 12 cm) with same sex littermates until testing at P90 ± 10 days. Food and water were available ad lib and a 12:12 light dark cycle was used. All animal protocols were performed with approval of the Nathan S. Kline Institute for Psychiatric Research Institutional Animal Care and Use Committee’s and were in accord with National Institutes of Health guidelines. Animal use followed the recommendations of the ARRIVE guidelines (Kilkenny et al., 2010).

### 2.2. Local field potential (LFP) recording and optical fiber implants

When the animals neared P90 they were implanted with either a single channel LFP telemetry device (model ETA-F10, DSI) with a 1 mm long bare electrode inserted in the frontal cortex (2.5 mm anterior to bregma, 2 mm lateral) to monitor sleep and wake bouts, or with a dual channel transmitter (model HD-X02, DSI) with a surface electrode in the frontal cortex and another in the ipsilateral visual cortex (4.0 mm posterior to Bregma, 3 mm lateral). Both spontaneous, and stimulus-evoked LFP data were digitized at 1,000 Hz and analyzed with Spike2 software. All animals also had bilateral optical fibers (Doric Lenses, MFC 200/250-0.66 ZF1.25(G) FLT) implanted in the frontal cortex at the coordinates of the LFP electrode.

### 2.3. Slow-wave sleep analyses

As previously described (Wilson et al., 2016), mice used for slow-wave sleep analyses were allowed to recover alone in their home cage for 3–5 days before 24-h recordings of basal sleep/wake cycling were begun. Data used for analysis began at 7 days post-surgery. Telemetry recordings do not require handling of the animals and continuous recordings post-surgery demonstrate normal sleep-wake cycles within 3 days. The transmitters used did not allow EMG measures, thus REM sleep was not monitored. All recordings were made from animals housed individually in sound attenuating chambers with developmentally ethanol-exposed and saline-exposed mice recorded simultaneously. Cortical LFP’s were acquired and digitized at 1,000 Hz and analyzed using Spike2 software (CED, Inc.). Slow-wave activity was identified by band-pass filtering for delta frequency (0.1–5 Hz) and root mean square (r.m.s.) delta amplitude extracted. Delta amplitude was determined in 14 s epochs and periods of high delta were identified as being at least 1 standard deviation above the mean r.m.s. amplitude over a given 24 h period. Artifacts were removed before mean and standard deviation calculations. The analyses of slow-wave bouts included the mean percent time in slow-wave over 24 h, mean slow-wave bout duration, and the mean number of slow-wave bout transitions/h of slow-wave activity. The accuracy of slow-wave sleep scoring was also confirmed in a small set of animals with simultaneous visual scoring over 1–2 h periods (*n* = 4) to ensure scored sleep epochs corresponded to behavioral sleep periods (Lewin et al., 2019).

### 2.4. Frontal cortex optical stimulation

Following 7 days of recovery, recordings and optogenetic manipulations were begun. Animals were placed in a chamber (14 cm wide × 30 cm long × 20 cm high) situated over the telemetry receiver for LFP recording and were connected to a 473 nm laser (Shanghai Laser and Optics Century Co.; model BL473T3H-100FC) *via* optical cables (Doric; SBP(2)200/230/900-0.57 2 m FCM-2xZF1.25) and an optical swivel (Doric; FRJ FC:FC) to allow free movement. Power output at the optical fiber tip was 8–10 mW. For assessment of SST-induced traveling cortical slow-waves, unilateral single pulse flashes (50 ms duration) were delivered at 20 s ISI with the animal freely moving (>200 repeats) and evoked responses recorded from both the site of the stimulus (frontal cortex) and simultaneously 6 mm posterior in the visual cortex. For closed loop stimulation triggered on spontaneous cortical slow-waves, the mice received bilateral 50 ms flash triggered on the frontal cortex LFP negative wave during slow-wave sleep at either 0 or 50 ms delay from the slow-wave trough nadir. Closed loop stimulation was controlled by a custom script in Spike2. The effect of this closed loop stimulation on on-going frontal cortex delta band oscillation amplitude was assessed with Fast-Fourier Transform [FFT] using 2 Hz bins during slow-wave bouts (>100 s periods) with and without optical stimulation. The temporal order of stimulation and no stimulation periods was counterbalanced across animals.

In addition to local field potential recordings in freely moving mice, SST-Cre × Ai32 mice (*n* = 4 saline and 4 ethanol exposed) were anesthetized with urethane (1 g/kg) and prepared for single-unit recording with an optotrode targeting the frontal cortex. Unit activity was recorded with a tungsten microelectrode (5 MOhms) attached to an optical fiber (200 μ). Single-unit activity was extracted with template matching (Spike2). Spontaneous activity and light-evoked activity (50 ms flash, 20 s ISI) were quantified with peristimulus time histograms, with attention paid to the early period of induced inhibition and the later excitation as previously described (Funk et al., 2017).

### 2.5. Histology

Following data collection, animals were perfused (PBS and 4% paraformaldehyde) and brains sectioned to identify electrode and optical fiber placements.

A separate set of mice were used to confirm P7 ethanol exposure effects on adult (>P90) SSTcre × Ai32 mice. Stereological counts of SST neuron number in neocortex compared 5 mice treated with P7 ethanol with 5 saline injected mice, using methods previously described (Smiley et al., 2015, 2019). At P90–110 mice were anesthetized by intraperitoneal injection of 200 mg/kg ketamine and 10 mg/kg xylazine, and transcardially perfused with heparinized 4% paraformaldehyde in phosphate buffer, pH 7.2. Brains were embedded in agar blocks for simultaneous sectioning and processing. The agar-embedded brains were cryoprotected by sinking in 20% buffered glycerol, prior to freezing and sectioning in coronal orientation at 50 μm thickness on a sliding microtome. A series of sections containing every 12th consecutive section through the cerebral cortex was process for double immunofluorescent labeling using chicken anti-GFP (1:500 dilution, Abcam, ab13970) that was visualized with Alexa Fluor 488 (Abcam, cat # 150173), and rabbit anti-somatostatin (1:1,000, Origen cat# AP33464SU-N) that was visualized by consecutive incubations in biotinylated goat anti-rabbit (Vectastain Cat# BA-1000) and streptavidin 594 (Invitrogen Cat# S11227).

Estimates of cell number used the Nv x Vref method (Gundersen et al., 1988). Labeled cells were sampled with a Basler acA5472 camera mounted on a motorized Nikon E600 microscope controlled with ImageJ software. Sections were systematically sampled at a grid of sites covering the left neocortex in each section. At each sampling site identically focused Z-stacks were obtained for the GFP- and somatostatin-immunolabels. Each Z-stack contained 6 images, with 3 μm spacing between images. Cell counting was done on optical disector counting boxes that were drawn on each Z-stack so that both labels could be simultaneously viewed, and counting boxes were 9 μm deep (3 Z-slices) with upper and lower guard zones of 3 and 6 μm, respectively. Each brain was sampled with 213 +/– 19 (mean +/– S.D.) sampling sites, and the coefficient of error for cell number (Dorph-Petersen et al., 2009) was 0.07 +/– 0.01. Average on-slide section thickness was 56 +/– 2 μm.

### 2.6. Data analyses

Data were analyzed using ANOVA and or *t*-tests, with repeated measures ANOVA’s used as appropriate. *Post hoc* comparisons were made with Fisher LSD tests. Significance was set at *p* < 0.05 for all tests.

## 3. Results

**FIGURE 1:**
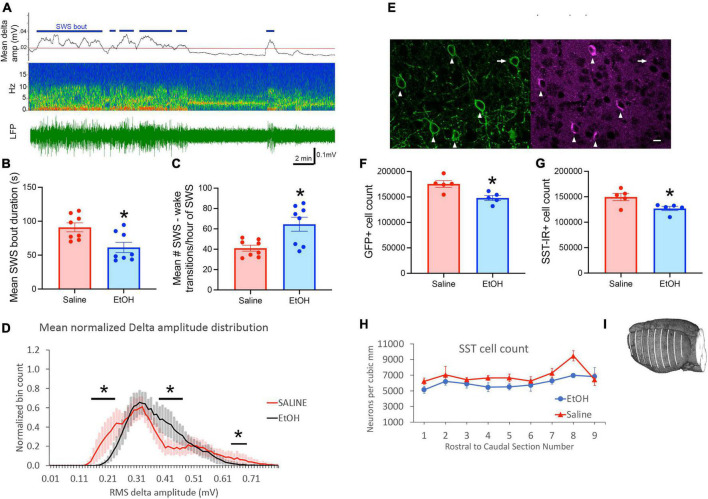
Slow-wave sleep in adult SST cre × Ai32 mice exposed to ethanol at P7 was impaired compared to saline controls. **(A)** Representative cortical LFP recording with raw LFP shown in bottom trace and time-frequency pseudocolor spectrograph shown in center. Top trace shows r.m.s. delta amplitude and 1 standard deviation above mean delta amplitude (red line) extracted from a 24 h recording. Periods of delta amplitude above the red line were classified as slow-wave sleep (horizontal blue markers) and coincided with behavioral inactivity as previously described (Wilson et al., 2016; Lewin et al., 2018). Slow-wave changes in ethanol mice compared to saline (*n* = 8/group) included **(B)** shorter SWS bout durations and **(C)** increased sleep-wake transitions, which together constitute sleep fragmentation. **(D)** In addition, delta amplitude oscillations were modified to display significantly fewer high amplitude waves. Horizontal lines highlight regions of significant *post hoc* comparisons between treatment groups (*p* < 0.05). **(E)** Stereological cell counts were used to evaluate the number of somatostatin cells in neocortex, in sections double labeled with ant-GFP and anti-somatostatin antibodies (*n* = 5 mice/group). In 85% of the SST-Cre cells, SST-immunolabeling (arrowheads) was found, but it was not confirmed in the remaining cells (arrow). We did not find SST-immunolabeled cells that lacked SST-Cre. Scale bar = 10 um. **(F,G)** P7 Ethanol exposure significantly reduced SST cell count as assessed in both GFP cells and SST immunolabeled cells. **(H)** Separation of stereological cell counts by anterior-posterior location indicated that the ethanol-induced reduction of SST cells is similar throughout the neocortex. Measured neuron densities were separated by their rostral-caudal section number. In brains that had more than 9 sections, the final small end sections were combined. This line graph provides a qualitative overview of local differences, as the stereological strategy was designed to sample the whole cortex, and data from each section includes an average of only 24 sampling sites from each brain. **(I)** The location of the neocortex (white lines) that was sampled for stereological estimates of SST neuron number and density is shown on a reconstruction of one brain made from block-face images taken during sectioning. Every 12th 50 uM thick coronal section through the neocortex was sampled. Asterisks signify significant difference between ethanol and saline conditions in all panels.

We first confirmed (*n* = 8/treatment group) that P7 ethanol induces similar effects on sleep in adult SST-Cre × Ai32 mice as previously reported in C57BL/6By mice (Wilson et al., 2016; Lewin et al., 2018; Apuzzo et al., 2020). As shown in Figure 1, SWS bout duration was significantly reduced [*t*(14) = 2.96, *p* = 0.01] and SWS transitions/hour of SWS were increased [*t*(14) = 3.15, *p* = 0.007] in P7 EtOH mice compared to saline controls. Cortical delta oscillation amplitude was also modified by P7 ethanol in a manner similar to that in C57BL/6By mice (Apuzzo et al., 2020) with a narrowing of the frequency distribution of delta amplitudes in ethanol mice (Figure 1D), including significantly reduced large amplitude oscillations [P7 exposure group × delta amplitude ANOVA, main effect of amplitude bin *F*(32,448) = 7.52, *p* < 0.0001] and exposure × bin interaction [*F*(32,448) = 1.49, *p* = 0.044]. Regions of significant *post hoc* differences are shown in Figure 1D. Large amplitude delta oscillations occur during slow-wave sleep thus the decrease in ethanol treated animals suggests an additional impact on sleep quality (Apuzzo et al., 2020).

In addition, we confirmed that SST neuron number was reduced in this mouse line by P7 ethanol. Stereological cell counts evaluated the reduction of somatostatin cells in whole neocortex caused by P7 ethanol treatment. Double labeling immunofluorescence identified somatostatin cells by GFP immunolabeling that was concentrated on the cell membrane, and somatostatin immunolabeling that was typically concentrated in cytoplasm of the cell soma (Figure 1E). Comparison of the two labels showed that about 85% of GFP cells had clearly distinguishable somatostatin-immunolabeling. Conversely, we did not find SST-immunolabeled cells that lacked SST-Cre. Comparison of ethanol and saline treated mice (*n* = 5 per treatment group) showed 16% reduction of GFP cells caused by ethanol treatment [Figure 1F, *t*(8) = 3.23, *p* = 0.01], and 15% reduction of SST-immunolabeled cells [Figure 1G, *t*(8) = 2.71, *p* = 0.027]. Separation of stereological cell counts by anterior-posterior section number (Figures 1H, I) indicated that the decrease of SST cell density was similar across cortex, as previously shown for parvalbumin and calretinin GABA cells after P7 ethanol treatment (Smiley et al., 2015).

**FIGURE 2:**
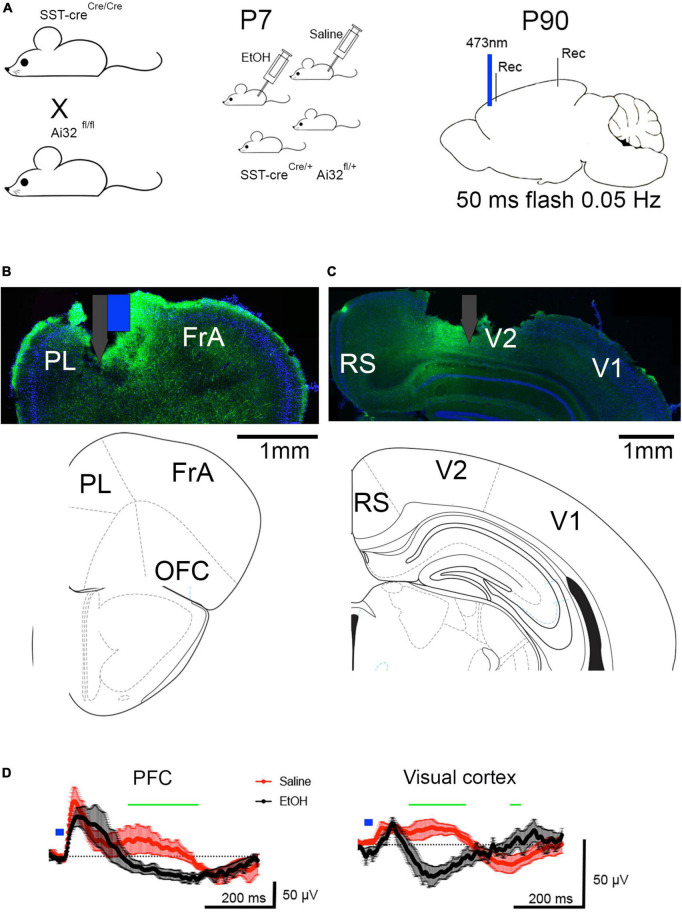
**(A)** Adult SSTcreXAi32 mice exposed to EtOH (*n* = 9) or saline (*n* = 5) at P7, received optical stimulation of SST neurons in prefrontal cortex. **(B)** Histological section showing typical prefrontal cortex optotrode location (including surface damage during histology). Associated atlas image below. White and Blue markers denote electrode (gray) attached to optical fiber (blue). PrL, prelimbic cortex; FrA, frontal association cortex, OFC, orbitofrontal cortex. Blue shows DAPI staining and green shows SST-GFP expression. **(C)** Histological and atlas section showing visual cortex electrode location (including surface damage during histology). RS, retrosplenial cortex, V1, primary visual cortex, V2, secondary visual cortex. **(D)** Prefrontal cortex optical stimulation evoked a robust, positive wave lasting 200–400 ms, which corresponds to a 2–5 Hz oscillation (delta frequency band) in P7 saline-treated adult mice and which traveled the anterior-posterior extent of the neocortex, producing a similar, though smaller wave in the visual cortex. The same stimulation in P7 ethanol-treated adult mice evoked an early field potential without the later evoked slow-wave. No evoked slow-wave was observed in the visual cortex of P7 ethanol-treated mice. Shown are means (solid line, *n* > 5 mice) and SEM. Blue mark indicates 473 nm, 50 ms flash in prefrontal cortex. Repeated measures ANOVA detects a significant difference between evoked waveforms in saline and ethanol treated mice at time points marked by green horizontal line. In PFC, the difference between saline and ethanol responses was primarily >200 ms post flash.

To examine the function of spared SST neurons in cortical circuit activity *in vivo* optogenetic stimulation protocols were used. Optical stimulation of PFC SST neurons (Figure 2A) evoked a response in the local field potential recorded in both the prefrontal cortex and the visual cortex (Figures 2B, C). The evoked potential in the visual cortex evoked by PFC activation is consistent with a rostral-caudal traveling slow-wave (Massimini et al., 2004; Niethard et al., 2018). In adults exposed to saline at P7 (*n* = 5), the evoked potential consisted of a short latency positive wave, followed by a lower amplitude, long-lasting (200–400 ms) positive wave in both the prefrontal and visual cortices. These potentials may reflect a synaptic inhibition and excitation sequence occurring in response to GABAergic inhibition evoked by the simultaneous activation of SST neurons (Funk et al., 2017). In contrast, in adults exposed to ethanol at P7 (*n* = 9), the long latency, prolonged positive evoked potential was absent in both the prefrontal and visual cortices (Figure 2D). Mean evoked potentials were significantly different between saline and ethanol treated animals in both the prefrontal cortex [ANOVA, time post-stimulus × P7 treatment interaction, *F*(300,2099) = 1.203, *p* = 0.0145] and the visual cortex [time × P7 treatment interaction, *F*(300,1798) = 4.877, *p* < 0.0001]. *Post hoc* Fisher comparisons revealed the differences were most pronounced during the period of the late positive wave. This suggests an impairment in SST neuron activation to induce slow-wave cortical oscillations in developmental ethanol exposed mice compared to saline exposed mice shown here, and as compared to previous reports (Funk et al., 2017).

**FIGURE 3:**
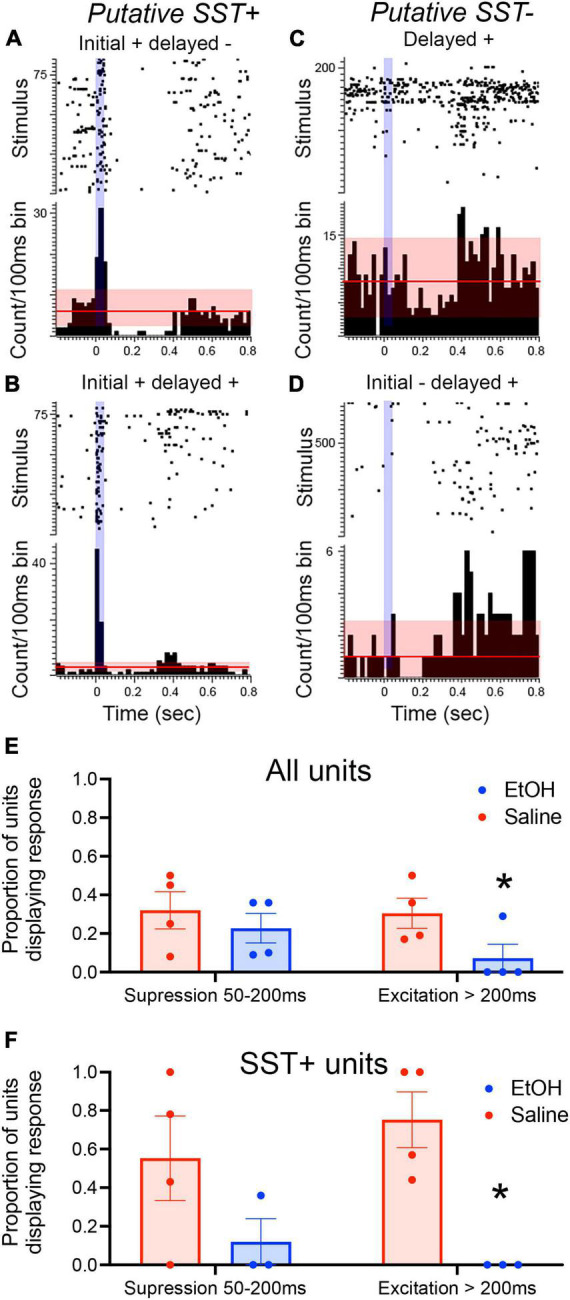
PFC unit recordings from adult SSTcreXAi32 mice exposed to P7 saline or ethanol (*n* = 4 saline mice, *n* = 47 units; 4 EtOH mice, *n* = 46 units). **(A–D)** Rasterplots and peri-stimulus time histogram examples of diverse unit responses to 50 ms, 473 nm light (vertical blue mark) in PFC of saline-treated mice. **(A)** A putative SST neuron in an ethanol exposed mouse excited during the light followed by suppression (relative to pre-stimulus activity). Horizontal line represents mean spontaneous activity with red shading representing ± 2 S.D. **(B)** A putative SST neuron in a saline-exposed mouse excited during the light and showing an excitatory rebound 300–400 ms later. **(C)** An ethanol-exposed non-SST neuron (i.e., no excitation to light) showing a delayed excitatory response 400–500 ms post flash. **(D)** A saline-exposed non-SST neuron displaying suppression 100–200 ms post-flash and an excitatory rebound at >400 ms. **(E)** Proportion of all units showing early (<200 ms) suppression and late (>200 ms) excitation in adults exposed to P7 saline or ethanol. There was a significant decrease in the probability of showing a late excitation response in the ethanol-treated mice (asterisk, p < 0.05). **(F)** Proportion of optogenetically identified putative SST neurons showing early (<200 ms) suppression and late (>200 ms) excitation in adults exposed to P7 saline or ethanol. There was a significant decrease in the probability of showing a late excitation response in the ethanol-treated mice (asterisk, *p* < 0.05) compared to saline controls.

As an initial assessment of the neural activity underlying these evoked potentials, single-unit activity in response to SST optical stimulation was examined in the PFC of urethane-anesthetized mice (*n* = 8 mice; 4 saline mice, *n* = 47 units; 4 EtOH mice, *n* = 46 units). As shown in Figures 3A, B, a subset of the units showed strong excitation during light stimulation, consistent with them being putative SST neurons. Immediately after light offset, about a third of all cells showed suppression which lasted < 200 ms, consistent with previous reports (Funk et al., 2017). In saline treated animals, a similar proportion also showed a late onset, rebound excitation which temporally coincides with the late positive wave recorded in the evoked potential. However, while units in P7 ethanol-exposed mice displayed a similar probability of initial suppression to the stimulus, significantly fewer cells showed the late onset excitation [*t*(6) = 2.187, *p* = 0.036], similar to the loss of the late evoked potential in ethanol-treated mice (Figure 3E). Similar analyses limited to putative SST neurons as determined by short latency excitation during light exposure (*n* = 20 saline, 13 EtOH) revealed similar effects of P7 ethanol [*t*(5) = 4.38, *p* = 0.004; Figure 3F]. There was no detectable difference in spontaneous activity between SST units recorded saline and ethanol-treated mice [saline mean = 1.48 ± 0.33 Hz, ethanol mean = 1.01 ± 0.25 Hz; *t*-test, *t*(29) = 1.06, *p* = 0.29]. These results suggest that the developmental ethanol-induced modification of cortical LFP slow wave oscillations is associated with an underlying impairment in SST single-unit activation.

**FIGURE 4:**
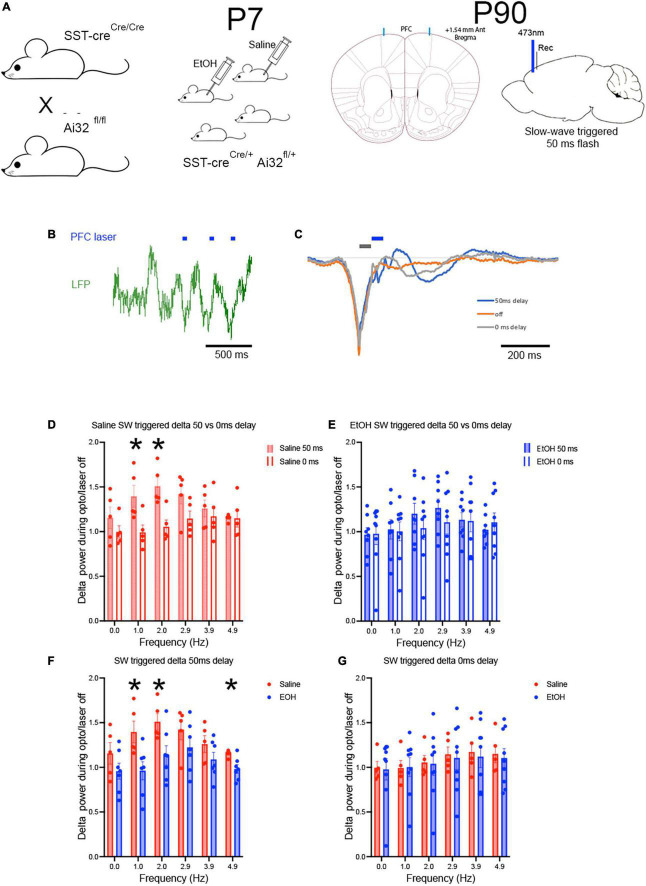
Using adult SSTcreXAi32 mice exposed to EtOH (*n* = 6) or saline at P7 (*n* = 6) **(A)**, closed loop optical stimulation of cortical SST GABAergic neurons in frontal cortex **(B)** with 50 ms flashes triggered on spontaneously occurring slow-waves, induced sustained slow wave activity in saline but not EtOH mice. Blue marks signify timing of light flashes. **(C)** A 50 ms delay in light activation reinforced slow-wave activity compared to no light, while a 0 ms delay was much less effective. Gray and blue marks signify timing of light flashes starting either 0 or 50 ms, respectively, post slow-wave sleep peak. **(D)** Over a prolonged period (30–60 min) of such stimulation, delta band oscillation amplitude was significantly enhanced in saline controls stimulated at the 50 ms delay but not at the 0 ms delay. Asterisks = sig. diff between delays. **(E)** In P7 EtOH treated adult mice, neither stimulation protocol was effective at enhancing delta oscillation amplitude. **(F,G)** Replotting the data shows the robust enhancement of delta amplitude in saline treated mice, but not in P7 EtOH treated mice, suggesting that stimulation of spared SST cortical neurons cannot compensate to improve sleep-related slow-wave amplitude. Asterisks = significant *post-hoc* test differences between groups.

Closed-loop stimulation triggered on cortical slow-waves can enhance cortical delta oscillations in humans and rodents (Ngo et al., 2013; Moreira et al., 2021). Similarly, direct modulation of cortical SST neuron activity can modify cortical slow-waves (Funk et al., 2017). Here, we used a closed loop optical stimulation paradigm to determine whether enhancing SST activity in-phase with spontaneous slow-waves could enhance delta oscillations and whether any such effect was impaired in mice with reduced numbers of SST neurons caused by P7 ethanol exposure (Figure 1; Smiley et al., 2019). Prefrontal cortex local field potentials were recorded near the implanted optical fiber in freely moving mice. Slow-waves were detected using voltage threshold to identify slow-wave troughs. Three different stimulation conditions were presented in a randomized order, block design wherein trough detection evoked a 50 ms flash either immediately or at a 50 ms delay, or no flash was evoked (Figure 4). The stimulation latencies were chosen based on preliminary data that showed a 50 ms delay evoked the strongest reinforcement of ongoing slow-wave activity in the evoked potential (Figure 4C).

In P7 saline exposed adult mice (*n* = 6), bilateral, prolonged (30–60 min), closed loop stimulation enhanced delta band oscillation power during the stimulation period compared to no stimulation periods before or after. As shown in Figure 4D, closed loop SST neuron stimulation at a 50 ms delay from the negative peak of spontaneous slow-waves significantly enhanced delta band power normalized to no stimulation [one-sample *t*-test, Saline normalized delta power was significantly greater than 1, *t*(5) = 5.218, *p* = 0.0034] and compared to stimulation at 0 ms delay [Saline 50 ms vs. 0 ms delta power, frequency × delay ANOVA, main effect of delay *F*(1,8) = 5.61, *p* = 0.045, frequency × delay interaction *F*(5,40) = 3.217, *p* = 0.015; *post-hoc* tests revealed significant difference in delta power enhancement between 0 and 50 ms delay]. However, the same SST stimulation protocol was ineffective in P7 ethanol-exposed adults [*n* = 6; Figure 4E; one-sample *t*-test, Ethanol normalized delta power was not significantly different from 1, *t*(5) = 1.309, *p* = 0.2474]. Neither stimulation latency produced a significant enhancement in delta power compared to no stimulation [Ethanol, 50 ms vs. 0 ms delta power, frequency × delay ANOVA, main effect of delay, *F*(1,15) = 0.099, *p* = 0.756; frequency × delay interaction, *F*(5,75) = 2.055, *p* = 0.081]. A direct comparison between the saline and ethanol P7 treatments (Figures 4F, G) revealed a significant difference between the exposure groups in the 50 ms delay protocol [frequency × treatment ANOVA, main effect of frequency, *F*(2.816,28.16) = 8.045, *p* = 0.0006; main effect of P7 treatment, *F*(1,10) = 5.101, *p* = 0.047; frequency × treatment interaction, *F*(5,50) = 1.99, *p* = 0.097]. There were no significant differences between saline and ethanol treatments with the 0 ms latency protocol. Importantly the effect of closed loop SST stimulation was selective for cortical delta power in these same animals. Analysis of mean normalized theta power (7–15 Hz) during slow-wave triggered optical stimulation detected no difference in theta power between saline (mean normalized change from no stimulation = 1.07 ± 0.07) and ethanol (mean = 1.02 ± 0.07) exposed mice [*t*-test, *t*(10) = 0.62, *p* = 0.55], nor any significant increase in normalized theta power compared to no stimulation control [saline, *t*(10) = 1.49, *p* = 0.17; ethanol, *t*(12) = 0.33, *p* = 0.74] (data not shown).

Together these results suggest a P7 ethanol exposure-induced impairment in neocortical SST neurons that may contribute to the known reduction in cortical delta oscillation amplitude and impaired sleep quality in these mice (Apuzzo et al., 2020).

## 4. Discussion

GABAergic neurons, including SST cells, are reduced in many structures by P7 ethanol exposure (Figure 1) (Smiley et al., 2015, 2019). These neurons play important roles in sleep physiology, which is also disrupted by P7 ethanol exposure (Figure 1) (Wilson et al., 2016; Lewin et al., 2018; Apuzzo et al., 2020). Through cell type-specific, optogenetic targeting of SST neurons in neocortex of saline control mice, we could evoke cortical slow-waves across the anterior-posterior axis of the cortex (Figure 2) and enhance delta band oscillation amplitude (Figure 4) during sleep as has previously been reported (Funk et al., 2017; Bugnon et al., 2022). However, in P7 ethanol exposed mice, the efficacy of this optogenetic manipulation was severely impaired, suggesting an SST neuron contribution to ethanol effects on slow-wave sleep cortical activity (Wilson et al., 2016; Apuzzo et al., 2020). More specifically, in saline control mice, selective stimulation of SST neurons in the PFC evoked a prolonged evoked potential and temporally coincident inhibition/excitation single-unit activity sequence (Figure 3) with time courses similar to a delta oscillation. This slow-wave evoked in the PFC propagated caudally to the visual cortex. Furthermore, closed-loop stimulation of PFC SST neurons in phase with spontaneous slow-waves enhanced delta band oscillation power during the stimulation. These results support the hypothesized role of SST neurons in cortical slow-wave oscillations that occur during non-REM sleep (Kuki et al., 2015; Funk et al., 2017; Niethard et al., 2018; Adamantidis et al., 2019; Bugnon et al., 2022), though do not exclude contributions of other cell types.

Postnatal-day seven ethanol exposure impaired SST neuron stimulation-induced evoked potentials, evoked potential propagation, single-unit late onset excitation, as well as the efficacy of closed loop SST stimulation to enhance cortical delta oscillations compared to saline controls. Together, these results support a significant contribution of developmental ethanol-induced SST neuron loss/dysfunction to at least one aspect of disrupted sleep physiology–abnormal cortical delta oscillations. It is not clear whether the impairments are due solely to SST cell loss, or whether SST cell function and connectivity are also modified. For example, while there was a 15% loss of cortical SST neurons following early ethanol, the large majority were spared. It is not clear however, whether the spared cells maintain normal network connectivity, excitability and/or pre- and post-synaptic strength. Cortical interneurons are capable of a variety of forms of plasticity (McFarlan et al., 2022). Specifically, the majority of cortical SST neurons are Martinotti cells (Funk et al., 2017; Bugnon et al., 2022) which have extensive axonal outputs targeting multiple layers but especially layer I where they could influence a relatively large population of pyramidal neurons (Wang et al., 2004; Funk et al., 2017). A decrease in the number or efficacy of these cells could decrease slow-wave amplitude by reducing the down-state magnitude and or synchrony. Slow-waves are propagated through cortico-cortical excitatory connections (Steriade et al., 1993a) although a subset of cortical SST also have long range projections (Tomioka et al., 2005; Funk et al., 2017). Again, a decrease in the number or efficacy SST cells in regions targeted by these connections could contribute to the reduced spread of slow waves as observed here in P7 ethanol treated adults. An examination of the effects of developmental ethanol exposure on cortical SST neuron axonal and dendritic elaboration and synaptic density, as well as cell excitability is warranted. Given the diverse contributions of cortical SST neurons in sleep, reduced or impaired SST neuron function may also contribute to other aspects of impaired sleep following early ethanol exposure, such as the observed decrease in sleep spindle density (Apuzzo et al., 2020).

That SST cells may contribute to modified delta oscillations in ethanol exposed mice suggests the possibility of artificially increasing the activity of spared SST neurons to improve cortical slow-wave amplitude. However, the impairment in closed-loop SST stimulation enhancement in delta oscillation amplitude observed here suggests that this type of manipulation may not be an effective treatment. Similar approaches in humans have revealed mixed results when using closed loop stimulation to enhance delta oscillations in elderly humans. For example, closed loop transcranial stimulation and auditory closed loop stimulation during slow wave sleep have both been shown to enhance slow-wave activity and memory consolidation in older adults (Ladenbauer et al., 2017; Papalambros et al., 2017). However, auditory closed loop stimulation was much less effective in older adults with mild cognitive impairment (Papalambros et al., 2019). Examination of more diverse closed-loop protocols (e.g., temporal patterns, intensity) in these different target populations may revealed maintained treatment efficacy in these populations.

Similar to SST neurons, parvalbumin (PV) expressing GABAergic neurons are also reduced (i.e., loss of neurons and/or protein expression) by early developmental ethanol exposure (Coleman et al., 2012; Smiley et al., 2015; Saito et al., 2019), however, post-exposure environment can reverse those effects (Apuzzo et al., 2020). Environmental enrichment can also reverse SST cell loss in cortex and hippocampus induced by developmental MK-801 (Murueta-Goyena et al., 2020), though the effects of this treatment on SST neurons after early ethanol exposure have not yet been tested. Given that postnatal environment can modulate many effects of developmental ethanol (Hannigan and Berman, 2000; Hamilton et al., 2014; Raineki et al., 2018) including reversing sleep impairment (Apuzzo et al., 2020), analysis of the effects of post-exposure environment on SST neurons and their sleep-related physiology is warranted.

Together, these findings implicate SST neuron loss as underlying at least one aspect of adult sleep dysfunction following developmental ethanol. Further identification of how different classes of SST (Yavorska and Wehr, 2016) and other GABAergic neurons mediate specific sleep related functions, and how these diverse cell types are affected by developmental ethanol may provide insight into possible targets for treatment of sleep impairment, and the resulting cascade of cognitive and emotional consequences following developmental EtOH.

## Data availability statement

The raw data supporting the conclusions of this article will be made available by the authors, without undue reservation.

## Ethics statement

This animal study was reviewed and approved by the Nathan S. Kline Institute for Psychiatric Research Institutional Animal Care and Use Committee.

## Author contributions

DW conceived of the project, designed the project, collected the data, analyzed the data, wrote manuscript draft and edited, and obtained the funding. GF and CW collected the data. CT provided the transgenic animal lines. JS analyzed the SST histology, discussed the project, edited the manuscript, and obtained the funding. MS discussed the project, edited the manuscript, and obtained the funding. All authors contributed to the article and approved the submitted version.
